# Clinical and demographic correlates of medication and visit adherence in a large randomized controlled trial

**DOI:** 10.1186/s12913-016-1471-x

**Published:** 2016-07-08

**Authors:** Jeff Whittle, José-Miguel Yamal, Jeffrey D. Williamson, Charles E. Ford, Jeffrey L. Probstfield, Barbara L. Beard, Horia Marginean, Bruce P. Hamilton, Pamela S. Suhan, Barry R. Davis, on behalf of the ALLHAT Collaborative Research Group

**Affiliations:** Clement J Zablocki Veterans Affairs Medical Center, Milwaukee, WI USA; The University of Texas School of Public Health, Houston, TX USA; Wake Forest University School of Medicine, Winston-Salem, NC USA; University of Washington Medical Center, Seattle, WA USA; E.A. Conway Hospital, Monroe, LA USA; Ottawa Hospital, Ottawa, ON Canada; Veterans Affairs Medical Center, Baltimore, MD USA; The University of Toledo, Toledo, OH USA; Division of Biostatistics, Coordinating Center for Clinical Trials, 1200 Pressler Street, Houston, TX 77030 USA

**Keywords:** Adherence, Race, Hypertension, Hyperlipidemia, Diuretics, Angiotensin-converting enzyme inhibitors, Calcium channel blockers

## Abstract

**Background:**

Patient characteristics are associated with adherence, which has implications for planning clinical research or designing payment systems that reward superior outcomes. It is unclear to what extent clinician efforts to improve adherence can attenuate these associations.

**Methods:**

To identify factors predicting visit and medication adherence in settings designed to optimize adherence, we did a retrospective analysis of participants in the Antihypertensive and Lipid-Lowering Treatment to Prevent Heart Attack Trial (ALLHAT). ALLHAT recruited participants at 632 sites in North America, Puerto Rico, and the U.S. Virgin Islands for random assignment to antihypertensive treatment with amlodipine, chlorthalidone, or lisinopril. Site investigators reported clinic characteristics at the time they applied to participate in the study and research coordinators used standardized methods to measure patient characteristics. We defined adequate visit adherence as attending at least 80 % of scheduled visits; adequate medication adherence was defined as taking 80 % or more of the randomly assigned medication at all study visits.

**Results:**

The 31,250 ALLHAT participants eligible for the visit adherence analysis attended 78.5 % of scheduled study visits; 68.9 % attended more than 80 % of scheduled visits. Clinic setting was predictive of both forms of adherence; adherence was worst at private clinics; clinics that enrolled more study participants had superior adherence. Adjusting for clinic characteristics and clinical factors, women, younger participants, Blacks and smokers were less likely to have adequate visit adherence. Among the 28,967 participants eligible for the medication adherence analysis, 21,261 (73.4 %) reported adequate medication adherence. In adjusted analyses, younger and less educated participants, Blacks, and smokers were less likely to report adequate adherence.

**Conclusions:**

Participant demographics were associated with adherence despite strenuous efforts to optimize adherence. Our results could inform decisions by researchers planning trials and policymakers designing payment systems.

**Trial registration:**

NCT00000542. Registered 27 October 1999.

**Electronic supplementary material:**

The online version of this article (doi:10.1186/s12913-016-1471-x) contains supplementary material, which is available to authorized users.

## Background

Adherence is defined as the extent to which a person’s behavior coincides with medical or health advice. This can refer to such diverse behaviors as personal habits (e.g., dietary changes) [1], attendance at scheduled visits (visit adherence) [2], and the extent to which patients take medication as prescribed [3].

Poor medication adherence has been associated with worse blood pressure control, worse clinical outcomes, and increased health care costs [4–8]. Proposals to link payment to such measures of quality as hypertension control have increased attention to mechanisms to enhance adherence. As participant adherence is considered an essential component of high-quality randomized clinical trials (RCT), efforts to ensure adherence have long been incorporated in RCT design [9, 10].

Extensive literature has examined factors associated with adherence in clinical practice. Medication adherence has been linked to gender, age, race, and ability to pay [11–15], as well as clinical factors such as the specific drug, dosing schedule, duration of therapy, and indication for therapy [11, 14]. However, many of these studies had serious flaws. Studies using administrative data often lacked detailed clinical information, in particular the indication for which the drug was prescribed. On the other hand, most studies that have used clinical data were smaller and performed in one or a few clinical settings, limiting generalizability. Finally, most studies were carried out in settings that did not have explicit strategies to enhance adherence, raising the question of whether greater use of these strategies could attenuate the association of participant characteristics with adherence.

Policymakers need to consider patient characteristics associated with poor adherence as they design payment systems that reward better outcomes; providers caring for populations with higher levels of poor adherence will be at risk for worse outcomes and therefore lower payments. Similarly, anticipating the likely level of adherence, particularly in population subgroups, is important for RCT design and conduct, as poor adherence to a therapy during a trial could obscure evidence of its efficacy. The evidence for a link between adherence and efficacy is well established for antihypertensive drug therapy [6, 16–18]. Therefore, we examined predictors of visit and medication adherence among individuals with hypertension who participated in a large RCT that used state-of-the-art methods to facilitate adherence.

## Methods

We performed secondary analysis of participants in the Antihypertensive and Lipid-Lowering Treatment to Prevent Heart Attack Trial (ALLHAT) [19, 20]. ALLHAT was a randomized trial of participants with hypertension who were aged 55 or older and at high risk for coronary artery disease events. The study explicitly selected sites able to enroll a diverse study population. Between February 1994 and May 1998, 632 clinics in the United States, Canada, Puerto Rico and the U.S. Virgin Islands recruited participants using mass mailings, media presentations, chart review, and word of mouth. The ALLHAT protocol was approved by the University of Texas Health Science Center at Houston Institutional Review Board (IRB) and all participants recruited into the study provided written informed consent. Each clinic received approval from its site IRB. For most participants, the study site was their primary care clinic. Participants received study drugs at no cost. Follow-up visits were scheduled at intervals established by study protocol; visit costs were not reimbursed. Key individuals at study clinics received ongoing feedback from regional coordinating centers regarding visit adherence and blood pressure control; they also received education regarding strategies to improve visit and medication adherence in regular conference calls, by written communication, and at annual investigator meetings; sites with exceptional performance were recognized at these meetings.

Participants were randomly assigned to 1 of 4 antihypertensive study drugs – amlodipine, chlorthalidone, lisinopril, or doxazosin. The study drug dose was adjusted regularly to achieve blood pressure control. Participants and providers were blinded to the identity of this drug. If blood pressure was uncontrolled on the study drug alone, additional antihypertensive drugs were added, following a study protocol. In the present analysis, we focus on adherence to the randomly assigned antihypertensive drug.

We included only participants who had at least five expected visits (i.e., 1 year of follow-up). We reasoned that the adherence of participants who did not complete a year in the study would not be representative of long term adherence. We also excluded participants randomized to receive doxazosin, as this arm was stopped early due to an excess of cardiovascular disease events and futility compared with chlorthalidone [21].

### Dependent variables

#### Visit adherence

The coordinating center maintained detailed records of attendance at follow-up visits scheduled at 1, 3, 6, 9, and 12 months following randomization; and every 4 months thereafter for up to 96 months. For most visits, there was a 4-month window during which a visit could occur and be considered adherent. During the first year, these windows were smaller – e.g., the 1 month visit had to occur between the day after randomization and 2 months following randomization. Although the maximum number of scheduled visits for a participant was 26, many participants were expected to have fewer visits due to death or later enrollment. To calculate a measure of visit adherence, we first divided the number of visits that were completed by the number of visits possible with perfect adherence. A dichotomous visit adherence variable was created for the primary analysis, with adherence considered to be poor if this proportion was <0.80 and adequate otherwise.

#### Medication adherence

At each follow-up visit, participants reported whether they had taken at least 80 % of their randomly assigned medication, a commonly used threshold for adequate compliance [6, 11, 22]. We analyzed medication adherence for participants who had attended at least five visits in which they were expected to be taking their randomized medication. We classified participants’ medication adherence as poor if they reported taking <80 % of their step 1 medication at any visit and as adequate otherwise.

### Predictor variables

When each site applied to participate in ALLHAT, the site investigator classified the clinical setting as private practice, group practice, staff model health maintenance organization (HMO), community health center, university, Veterans Affairs Medical Center (VAMC), or other. We characterized clinics by the number of ALLHAT patients they enrolled, then divided participants into ten equal groups, based on the enrollment volume of their clinic; participants in the first decile attended clinics enrolling 1 to 33 participants, those in the tenth, clinics enrolling 492–607.

At the time of randomization, study coordinators collected data elements that we hypothesized might be associated with adherence. These included age, gender, race, ethnicity and education, as well as several clinical variables. We categorized age as 55–64, 65–74, and >74 years of age. In sensitivity analyses, we repeated the analysis, first treating age as a continuous variable and then using 5 year cutpoints; results were similar, so we report only the original categorization. We categorized self-reported years of education as < 12 years, 12 years, or > 12 years. At the time of randomization, the site coordinator categorized each participant’s self-reported race as White, Black, American Indian/Alaskan Native, Asian/Pacific Islander, or other,. In addition, the coordinator asked whether the participant was of Hispanic origin; response options were yes, no, and don’t know. Because some prior studies have found that Black race and Hispanic ethnicity are associated with lower adherence, we analyzed Blacks as a single group, regardless of Hispanic origin and divided non-Blacks into those who self-identified as Hispanic and those who did not.

We measured overall baseline health with the question, “In general, would you say your health is excellent, very good, good, fair, or poor?” We used medical record review at the time of randomization to establish the presence or absence of diabetes mellitus, HDL cholesterol < 35 mg/dl, subclinical atherosclerotic cardiovascular disease, and atherosclerotic cardiovascular disease. We categorized a participant as possibly disabled if they were <65 years old and had Medicare insurance. We also examined baseline characteristics that suggested more or less concern about health issues, including cigarette smoking (classified as never, past, or current), daily aspirin use, and obesity (body mass index ≥ 30 kg/m^2^). Finally, we considered how long participants had been treated for their hypertension, a surrogate for when the condition had been recognized.

### Statistical analysis

For our primary analyses we first compared the proportion of participants with adequate visit and medication adherence across categories of each predictor variable using chi-square tests or Fisher’s exact test, as appropriate. We then used multivariable logistic regression to identify baseline characteristics independently associated with adherence. For each type of adherence, we entered all baseline variables into the regression model as covariates, then performed stepwise backward variable selection (probability 0.10 to remove a variable and 0.05 for reentry). We confirmed goodness of fit using the Pearson chi-square test.

In a secondary analysis of visit adherence, we used the continuous form of visit adherence (proportion of expected visits attended) after an arcsine (square root) transformation for its non-normal distribution. In this analysis, our multivariable analysis used linear regression. We did not perform a similar analysis of a continuous measure of medication adherence, as this variable was so non-normally distributed.

In sensitivity analyses, we excluded visits after a participant developed cancer (except non-melanoma skin cancer) or end stage renal disease (ESRD) or had a new cardiovascular disease event, including stroke, myocardial infarction, coronary artery revascularization, angina, congestive heart failure, or peripheral vascular disease, because it is possible that patterns of adherence would change after such events. We used STATA version 12.0 (StataCorp LP, College Station, TX) for all statistical analyses.

## Results

The ALLHAT study achieved excellent ethnic and gender diversity (Table 1). While 40 % of participants were enrolled from the southern United States, the population was also geographically diverse; Puerto Rico and the Virgin Islands together contributed 13 % of enrollees.

**Table 1:** Baseline characteristics by medication and visit adherence

Baseline characteristic	Medication adherence*			Visit adherence^†^		
	N	Adequate adherence	*P*-value	N	Adequate adherence	*P*-value
Gender, n (%)			<0.001			<0.001
Male	16,344	11,942 (73.1)		17,343	12,464 (71.9)	
Female	14,020	9,955 (71.0)		15,395	9,656 (62.7)	
Age group, n (%)			<0.001			<0.001
55–64	13,037	9,481 (72.7)		14,014	9,191 (65.6)	
65–74	12,250	8,885 (72.5)		13,176	9,152 (69.5)	
≥75	5,077	3,531 (69.5)		5,548	3,777 (68.1)	
Race and Ethnicity, n (%)			<0.001			<0.001
Black	10,616	7,269 (68.5)		11,572	6,950 (60.1)	
Non-Black Hispanic	4,377	2,772 (63.3)		5,146	2,784 (54.1)	
Non-Black Non-Hispanic	15,371	11,856 (77.1)		16,020	12,386 (77.3)	
Education, n (%)			<0.001			<0.001
< High school	12,076	8,340 (69.1)		13,215	8,530 (64.5)	
High school	8,194	6,068 (74.1)		8,700	6,157 (70.8)	
Beyond high school	8,138	6,137 (75.4)		8,609	6,263 (72.7)	
Type II Diabetes, n (%)			0.721			<0.001
Yes	10,879	14,065 (72.2)		11,810	7,757 (65.7)	
No	19,485	7,832 (72.0)		20,928	14,363 (68.6)	
Smoking, n (%)			<0.001			<0.001
Current	6,596	4,673 (70.8)		7,121	4,657 (65.4)	
Past	12,395	9,112 (73.5)		13,212	9,462 (71.6)	
Never	11,372	8,111 (71.3)		12,403	8,001 (64.5)	
Self-assessed baseline health, n (%)			<0.001			<0.001
Excellent	1,735	1,312 (75.6)		1,813	1,332 (73.5)	
Very good	7,090	5,249 (74.0)		7,639	5,415 (70.9)	
Good	13,490	9,747 (72.3)		14,593	9,873 (67.7)	
Fair	6,905	4,748 (68.8)		7,440	4,790 (64.4)	
Poor	837	609 (72.8)		917	530 (57.8)	
Unknown	307	232 (75.6)		336	180 (53.6)	
Practice type, n (%)			<0.001			<0.001
Private	8,895	5,867 (66.0)		9,845	6,169 (62.7)	
Group	6,035	4,705 (78.0)		6,403	4,621 (72.2)	
HMO	872	690 (79.1)		1,150	567 (49.3)	
Community Health Center	2,519	1,796 (71.3)		2,663	1,674 (62.9)	
University	2,878	2,060 (71.6)		3,011	2,195 (72.9)	
Other	2,530	1,940 (76.7)		2,744	1,795 (65.4)	
VAMC	5,340	3,924 (73.5)		5,503	4,541 (82.5)	
Unknown	1,295	915 (70.7)		1,419	558 (39.3)	
Clinic enrollment volume, n (%)			<0.001			<0.001
1–33 participants	3135	2086 (66.5)		3299	2077 (63.0)	
34–54 participants	3161	2332 (73.8)		3318	2262 (68.2)	
55–74 participants	3220	2404 (74.7)		3388	2389 (70.5)	
75–100 participants	3255	2269 (69.7)		3393	2497 (73.6)	
101–125 participants	2865	2233 (77.9)		3008	2126 (70.7)	
126–170 participants	3104	2196 (70.7)		3339	2276 (68.2)	
171–215 participants	3176	2392 (75.3)		3313	2570 (77.6)	
216–325 participants	2940	2310 (78.6)		3354	2307 (68.8)	
326–491 participants	2833	2044 (72.1)		3360	1738 (51.7)	
492–607 participants	2675	1631 (61.0)		2966	1878 (63.3)	
Baseline medication use, n (%)			<0.001			<0.001
On drug therapy ≥ 2 months	26,375	19,174 (72.7)		28,416	19,486 (68.6)	
On drug therapy < 2 months	1,025	701 (68.4)		1,114	684 (61.4)	
Currently untreated	2,964	2,022 (68.2)		3,207	1,950 (60.8)	
HDL cholesterol < 35 mg/dl, n (%)			<0.001			<0.001
Yes	3,608	2,732 (75.7)		3,829	2,842 (74.2)	
No	26,756	19,165 (71.6)		28,909	19,278 (66.7)	
Aspirin use, n (%)			<0.001			<0.001
Yes	11,053	8,300 (75.1)		11,737	8,654 (73.7)	
No	18,955	13,348 (70.4)		20,591	13,260 (64.4)	
Geographic region, n (%)			<0.001			<0.001
Northeast	4,637	3,399 (73.3)		4,918	3,489 (70.9)	
Midwest	5,704	4,141 (72.6)		5,932	4,510 (76.0)	
South	12,799	9,471 (74.0)		13,624	8,963 (65.8)	
West	3,045	2,183 (71.7)		3,164	2,408 (76.1)	
Canada	537	422 (78.6)		542	509 (93.9)	
Puerto Rico/Virgin Islands	3,642	2,281 (62.6)		4,558	2,241 (49.2)	
Possible disability, n (%)			0.712			<0.001
Yes	5,533	3,979 (71.9)		5,836	4,414 (75.6)	
No	24,831	17,918 (72.2)		26,902	17,706 (65.8)	
ASCVD, n (%)			<0.001			<0.001
Yes	10,965	8,145 (74.3)		11,863	8,270 (69.7)	
Sub-clinical	8,487	5,749 (67.7)		9,029	6,047 (67.0)	
No	10,912	8,003 (73.3)		11,846	7,803 (65.9)	
BMI, n (%)			0.029			0.040
≤30	17,602	12,607 (71.6)		19,014	12,939 (68.1)	
>30	12,672	9,221 (72.8)		13,624	9,124 (67.0)	
Randomized drug, n (%)			0.028			<0.001
Chlorthalidone	13,890	10,075 (72.5)		14,975	10,255 (68.5)	
Amlodipine	8,275	6,002 (72.5)		8,875	6,094 (68.7)	
Lisinopril	8,199	5,820 (71.0)		8,888	5,771 (64.9)	

*Medication Adherence is defined as poor adherence if the patient reported taking “Less than 80 % of the Step 1 medication” at any visit and adequate adherence otherwise

†Visit Adherence is defined as poor adherence if the patient attended < 80 % of scheduled visits within the study window and adequate adherence if they attended  ≥ 80 % of scheduled visits within the study window

### Visit adherence

Of 33,357 ALLHAT participants randomly assigned to amlodipine, chlorthalidone, or lisinopril, 32,738 were eligible for the visit adherence analysis. Overall, they attended 78.2 % of expected visits; 22,120 (67.6 %) had adequate adherence (the percentage of visits attended ≥ 80 %). Participants with adequate visit adherence were more likely to be male, older, have more than a high school education, and be of non-Hispanic, non-Black ethnicity. They reported themselves as having better health, and indeed had less clinical cardiovascular disease, but were no less likely to have diabetes mellitus and were more likely to have HDL cholesterol < 35 mg/dl. Our measures of health awareness suggested they were more attentive to their health; they were more likely to have been on hypertension treatment for more than 2 months, take aspirin, have quit smoking, and be non-obese. They were more likely to be treated in the US or Canada, in a VAMC, HMO or group practice site, at a clinic that enrolled more participants, and not be randomized to receive lisinopril.

In multivariable analysis, the demographic factors associated with adequate adherence tended to remain significant (Table 2), except educational level became non-significant and both past- and never-smokers were more adherent. Among clinical factors, better self-reported health, subclinical cardiovascular disease, and low HDL – but not diabetes –were associated with adequate adherence. Factors we considered to represent health awareness, with the exception of obesity, continued to be associated with adherence. The clinic site factors associated with adherence changed after adjustment for demographic and clinical factors. While clinic enrollment volume continued to be associated with better adherence, geographic location was no longer significant; after adjustment, group practice, university, and VAMC clinics were associated with better adherence and HMO clinics with worse visit adherence.

**Table 2:** Independent effect of baseline characteristics on odds of adequate visit adherence^a^

	Odds ratio (95 % CI) - Multiple logistic regression			
Baseline characteristic	Full model		Reduced model	
	(*N* = 30,262)	*P*-value	(*N* = 30,262)	*P*-value
Male	1.07 (1.01–1.13)	0.033	1.07 (1.01–1.13)	0.029
Age (55–64 is reference group)				
65–74	1.38 (1.30–1.48)	<0.001	1.37 (1.29–1.46)	<0.001
≥75	1.34 (1.24–1.46)	<0.001	1.32 (1.22–1.43)	<0.001
Race and Ethnicity (Black is reference group)				
Non-Black Hispanic	0.94 (0.87–1.02)	0.126	0.94 (0.87–1.01)	0.100
Non-Black Non-Hispanic	1.98 (1.86–2.11)	<0.001	2.00 (1.88–2.13)	<0.001
Education (High school is reference group)				
< High school	0.96 (0.90–1.02)	0.195		
Beyond high school	1.01 (0.94–1.09)	0.741		
Has Diabetes	0.92 (0.87–0.98)	0.010	0.92 (0.87–0.98)	0.010
Smoking (Current is ref group)				
Past	1.19 (1.10–1.28)	<0.001	1.19 (1.11–1.29)	<0.001
Never	1.12 (1.04–1.20)	0.004	1.12 (1.04–1.21)	0.003
Self-assessed baseline health (Excellent is reference group)				
Very good	0.91 (0.80–1.03)	0.137	0.91 (0.80–1.03)	0.131
Good	0.89 (0.79–1.00)	0.050	0.88 (0.78–1.00)	0.042
Fair	0.80 (0.70–0.91)	<0.001	0.79 (0.70–0.90)	<0.001
Poor	0.52 (0.43–0.62)	<0.001	0.51 (0.43–0.61)	<0.001
Unknown	0.61 (0.42–0.89)	0.010	0.60 (0.41–0.88)	0.009
Practice type (Private is reference group)				
Group	1.23 (1.13–1.33)	<0.001	1.23 (1.14–1.33)	<0.001
HMO	0.58 (0.51–0.66)	<0.001	0.58 (0.51–0.66)	<0.001
Community Health Center	1.04 (0.95–1.15)	0.406	1.04 (0.94–1.15)	0.434
University	1.56 (1.41–1.73)	<0.001	1.56 (1.41–1.72)	<0.001
Other	1.15 (1.04–1.27)	0.008	1.15 (1.04–1.27)	0.007
VAMC	2.00 (1.82–2.19)	<0.001	2.00 (1.82–2.20)	<0.001
Unknown	0.41 (0.37–0.47)	<0.001	0.41 (0.37–0.47)	<0.001
Baseline medications (Untreated is reference group)				
On drug therapy < 2 months	1.41 (1.30–1.54)	<0.001	1.42 (1.30–1.54)	<0.001
On drug therapy ≥ 2 months	1.10 (0.94–1.28)	0.233	1.10 (0.94–1.28)	0.222
HDL Cholesterol < 35 mg/dl	1.09 (1.00–1.19)	0.052	1.09 (1.00–1.19)	0.048
Taking aspirin	1.20 (1.13–1.27)	<0.001	1.20 (1.13–1.27)	<0.001
Possible disability	2.04 (1.88–2.21)	<0.001	2.03 (1.87–2.20)	<0.001
ASCVD (No ASCVD is reference group)				
Yes	0.90 (0.84–0.97)	0.003	0.90 (0.84–0.96)	0.003
Sub–clinical	1.03 (0.96–1.11)	0.447	1.03 (0.95–1.11)	0.483
BMI > 30	1.02 (0.97–1.08)	0.453		
Decile of clinic enrollment volume	1.03 (1.02–1.04)	<0.001	1.03 (1.02–1.04)	<0.001
Randomized treatment group (Chlorthalidone is reference group)				
Amlodipine	1.01 (0.95–1.07)	0.774	1.01 (0.95–1.07)	0.782
Lisinopril	0.84 (0.79–0.90)	<0.001	0.84 (0.79–0.90)	<0.001

^a^Visit Adherence is a dichotomous variable defined as poor if the patient’s “% visit adherence” was < 80 % and adequate if adherence was ≥ 80 %

### Medication adherence

There were 30,364 ALLHAT participants who attended five or more visits where they were still taking their randomly assigned medication. In Table 1 we compare the baseline characteristics of the 21,897 (72.1 %) who reported taking 80 % or more of their randomized medication at each of their study visits to those who reported taking less than 80 % at 1 or more visits. The pattern of bivariable comparisons are similar to those we found for visit adherence, except that younger participants were more, rather than less, likely to be medically adherent, and diabetes mellitus was not associated with medical adherence.

In multivariable analysis, in contrast to visit adherence, age was inversely associated with adequate medication adherence, while gender was not significant. Non-Hispanic non-Blacks had better medication and visit adherence than Blacks or Hispanic non-Blacks. The impacts of randomization to lisinopril treatment group and educational status were small but still significant. The impact of clinical factors was generally less than with visit adherence, and the directionality was often different. Thus, individuals with subclinical cardiovascular disease were less likely to be medically adherent, the impact of self-reported health status was small, though significant, and diabetes mellitus was not significant. Our measures of health awareness were associated with medical adherence in the same pattern as with visit adherence: nonsmoking, aspirin use, and prior treatment of hypertension predicted better adherence but obesity had no effect. As with visit adherence, higher clinic enrollment volume was associated with better medication adherence, but other associations with clinic type were markedly different (Table 3). Visit adherence was not associated with medication adherence.

**Table 3:** Independent effect of baseline characteristics on medication adherence^a^

	Odds ratio (95 % CI) - Multiple logistic regression			
	Full model		Reduced model	
Baseline characteristic	(*N* = 28,176)	*P*-value	(*N* = 28,176)	*P*-value
Adequate Visit Adherence	0.97 (0.91–1.03)	0.330		
Male	1.01 (0.94–1.07)	0.885		
Age (55–64 is the ref group)				
65–74	0.92 (0.85–0.98)	0.014	0.91 (0.85–0.97)	0.007
≥75	0.82 (0.75–0.89)	<0.001	0.81 (0.74–0.88)	<0.001
Race and Ethnicity (Black is reference group)				
Non-Black Hispanic	0.76 (0.69–0.82)	<0.001	0.75 (0.69–0.82)	<0.001
Non-Black Non-Hispanic	1.45 (1.36–1.55)	<0.001	1.45 (1.36–1.55)	<0.001
Education (High school is the reference group)				
< High school	0.91 (0.85–0.97)	0.006	0.91 (0.85–0.97)	0.006
Beyond high school	1.02 (0.95–1.09)	0.645	1.02 (0.95–1.09)	0.654
Has diabetes	0.96 (0.90–1.02)	0.207		
Smoking (Current is reference group)				
Past	1.13 (1.05–1.22)	0.001	1.13 (1.05–1.21)	0.001
Never	1.15 (1.06–1.24)	<0.001	1.14 (1.06–1.23)	<0.001
Self-assessed baseline health (Excellent is reference group)				
Very good	0.93 (0.82–1.06)	0.283	0.93 (0.82–1.06)	0.287
Good	0.90 (0.80–1.02)	0.096	0.90 (0.80–1.02)	0.093
Fair	0.79 (0.70–0.90)	<0.001	0.79 (0.70–0.90)	<0.001
Poor	0.94 (0.77–1.14)	0.515	0.94 (0.77–1.14)	0.509
Unknown	0.89 (0.59–1.35)	0.590	0.89 (0.59–1.34)	0.568
Practice type (Private is reference group)				
Group	1.56 (1.44–1.70)	<0.001	1.56 (1.44–1.70)	<0.001
HMO	2.00 (1.68–2.39)	<0.001	2.01 (1.68–2.40)	<0.001
Community Health Center	1.22 (1.10–1.36)	<0.001	1.22 (1.10–1.36)	<0.001
University	1.22 (1.10–1.35)	<0.001	1.21 (1.10–1.34)	<0.001
Other	1.70 (1.52–1.90)	<0.001	1.70 (1.52–1.90)	<0.001
VAMC	1.18 (1.08–1.29)	<0.001	1.18 (1.08–1.28)	<0.001
Unknown	1.46 (1.28–1.68)	<0.001	1.48 (1.29–1.70)	<0.001
Baseline Medications (Untreated is reference group)				
On drug therapy < 2 months	1.24 (1.13–1.35)	<0.001	1.23 (1.13–1.35)	<0.001
On drug therapy ≥ 2 months	1.11 (0.94–1.30)	0.225	1.11 (0.94–1.30)	0.224
HDL Cholesterol < 35 mg/dl	1.03 (0.94–1.12)	0.585		
Taking aspirin	1.14 (1.07–1.21)	<0.001	1.13 (1.07–1.20)	<0.001
Possible disability	0.91 (0.84–0.99)	0.033	0.91 (0.84–0.99)	0.022
ASCVD (No ASCVD is reference group)				
Yes	0.94 (0.87–1.01)	0.082	0.95 (0.89–1.01)	0.117
Sub-clinical	0.76 (0.71–0.82)	<0.001	0.77 (0.72–0.83)	<0.001
BMI > 30	1.05 (0.99–1.11)	0.105		
Decile of clinic enrollment volume	1.05 (1.04–1.06)	<0.001	1.05 (1.04–1.06)	<0.001
Randomized treatment group (Chlorthalidone is reference group)				
Amlodipine	0.99 (0.93–1.05)	0.722	0.99 (0.93–1.05)	0.722
Lisinopril	0.92 (0.86–0.98)	0.010	0.92 (0.86–0.98)	0.012

^a^Medication adherence is a dichotomous variable defined as poor adherence if participants reported taking less than 80 % of their step 1 medication at any visit and as good if they reported taking 80 % or more of this medication at all visits

### Sensitivity analyses

The predictors of visit adherence were unchanged when visit adherence was treated as a continuous variable (see Additional file 1: Table S1, which shows baseline characteristics by continuous visit adherence). The factors associated with adherence were also similar when we repeated our analysis excluding visits after a participant developed ESRD or cancer, or suffered a cardiovascular disease event, except randomization to lisinopril was not associated with medication adherence and diabetes mellitus was not associated with visit adherence (see Additional file 1: Tables S2-S4, which show the unadjusted and adjusted predictors of adherence with truncation after these events).

## Discussion

In this large diverse cohort with structured follow-up for hypertension at clinics that received audit and feedback similar to current “best practices” for chronic disease management [23, 24], we found that clinical and demographic participant characteristics remained associated with both visit and medication adherence. While these associations are not large enough to aid clinical decision-making for individual patients, they could significantly affect reimbursement in systems that reward improved adherence. Similarly, these differences could have important implications for designing and powering RCTs. Moreover, while clinic characteristics impact both medication and visit adherence, the ability of larger, more structured clinics (e.g., VAMC, university, or group practices) to achieve better visit adherence did not translate into improved medication adherence. Indeed, better visit adherence was not associated with improved medication adherence at the participant level, suggesting that closer follow-up alone may not improve medication adherence.

The patterns of medication adherence that we observed are consistent with patterns of adherence seen in settings with fewer resources to support adherence. Most, but not all, studies have found lower adherence among Blacks and Hispanic non-Blacks [15, 25–28] and persons with less education [29]. The association of adherence with age and gender has been less consistent across studies [11]. Although the literature is not as extensive, demographic characteristics have also been associated with visit adherence [3, 30].

In contrast to prior non-randomized studies suggesting better medication adherence among persons receiving angiotensin-converting enzyme inhibitors, we observed decreased medication and visit adherence among participants randomized to lisinopril [31–33]. However, this association lost significance when we excluded data following clinical events that may have decreased adherence. We note that the lisinopril group had higher rates of strokes, combined cardiovascular disease, and heart failure [19], which may have affected adherence. Further studies should confirm these findings in other settings.

We acknowledge that our findings are most easily generalized to other RCTs. Individuals who participate in RCTs are known to be more adherent than the general population [9]. Similarly, the clinics which participate in randomized trials may systematically differ from the universe of clinics. These factors suggest we may have overestimated adherence. It is also possible the effects of participant variables are attenuated, since these participants may be more uniformly interested in health, causing factors such as race, age, and gender to have less impact on adherence. The same might apply to clinics – the federally-qualified health clinics and VAMCs that participated in this study, for example, may be more like one another than federally-qualified health clinics and VAMCs in general. Thus, the odds ratios we found may be conservative estimates of the impact of patient and clinic characteristics on adherence. Similarly, since ALLHAT began in 1994, with the last closeout visits happening in 2002, it is possible that factors influencing adherence have changed since that time, although the techniques used to support adherence in ALLHAT continue to be viewed as best practices [23, 24]. We acknowledge this may affect extrapolation of our results to current practice.

We also note that we studied adherence in the setting of hypertension, a chronic disease, using drugs from just three classes; our findings may not apply to other disease/medication dyads [28]. Moreover, medication adherence may have been affected by the fact that doctor and participant were unaware of which medication they were using. Finally, our choice of measures of visit and medication adherence influenced our results. Our generous 4 month window for considering a visit “adherent” likely inflates our visit adherence compared to other reports. Our single item self-report measure of medication adherence might give different results than analyses based on more complex self-report measures [17, 34], measures of medication refill behavior [22], or electronic pill counters [35].

Despite these limitations, we believe the strengths of the study make the results noteworthy for clinical practice and policy. We studied adherence in a large, diverse population treated in a wide variety of clinical settings, using standardized measures of visit and medication adherence. Participants and sites used the same drugs on the same schedule for the same indication and received similar support for medication and visit adherence – patients received regular monitoring, reminder contacts, and free prescriptions; clinics received audit and feedback, typically had dedicated staff and were recognized for high performance. All these factors reduce variation due to local clinic policies or clinician choices.

## Conclusions

We believe our findings suggest three considerations regarding adherence that may apply to patient care. First, adherence is unpredictable. Although patient characteristics are significantly associated with adherence, they do not identify patient groups with uniformly adequate medication adherence. For example, while the odds of adequate medication adherence among non-Hispanic, non-Blacks were nearly 50 % higher than among Blacks, large majorities of both non-Hispanic, non-Blacks, and Blacks were adherent; significant minorities of both groups were not. Interventions targeting patients at particular risk of poor adherence ignore this fact. Second, patients who come to visits more regularly should not be assumed to take their medications routinely; in our analysis, visit adherence did not predict medication adherence. Finally, poor adherence may persist even in well-run clinics with motivated patients. This suggests that we may still need new approaches to improving adherence. These results from the largest RCT of hypertensive therapy also confirm that study designers must attend to participant factors associated with adherence to ensure adequate power to detect significant differences, particularly in important subgroups.

On the policy level, if our results can be extrapolated to current practice, then they suggest that systematic variation in adherence by patient characteristics will persist despite vigorous efforts to support adherence. This would buttress prior concerns that clinics serving certain populations will be disadvantaged if financial rewards or penalties are distributed based on adherence [36, 37].

## Abbreviations

ALLHAT, antihypertensive and lipid-lowering treatment to prevent heart attack trial; HMO, Health Maintenance Organization; IRB, institutional review board; RCT, randomized clinical trial; VAMC, Veterans Affairs Medical Center

## Additional file

Additional file 1: It contains 4 supplementary tables entitled, respectively: Table S1. Baseline Characteristics by Continuous Visit Adherence*. Table S2. Baseline Characteristics by Medication and Visit Adherence, Truncated after Event. Table S3. Independent Effect of Baseline Characteristics on Visit Adherence, Truncated after Event. Table S4. Independent Effect of Baseline Characteristics on Medication Adherence, Truncated after Event. These tables contain additional detail regarding the study results. Table S1 provides an alternative analysis of the bivariable association between participant characteristics and visit adherence. Tables S2–S4 present the results of sensitivity analyses. (DOCX 39 kb)
