# Supplementary material for: Clinical and demographic correlates of medication and visit adherence in a large randomized controlled trial
Source: BMC Health Serv Res. 2016 Jul 8;16:236. doi: 10.1186/s12913-016-1471-x (PMC4938977; doi:10.1186/s12913-016-1471-x)
Supplement: Additional file 1: — It contains 4 supplementary tables entitled, respectively: Table S1. Baseline Characteristics by Continuous Visit Adherence*. Table S2. Baseline Characteristics by Medication and Visit Adherence, Truncated after Event. Table S3. Independent Effect of Baseline Characteristics on Visit Adherence, Truncated after Event. Table S4. Independent Effect of Baseline Characteristics on Medication Adherence, Truncated after Event. These tables contain additional detail regarding the study results. Table S1 provides an alternative analysis of the bivariable association between participant characteristics and visit adherence. Tables S2–S4 present the results of sensitivity analyses. (DOCX 39 kb) [file 12913_2016_1471_MOESM1_ESM.docx]

**Additional file 1**

**Supplement Digital Content**

**Clinical and Demographic Correlates of Medication and Visit Adherence
in a Large Randomized Controlled Trial**

Jeff Whittle, MD, MPH^*^; José-Miguel Yamal, PhD^†^; Jeffrey D. Williamson, MD, MHS^‡^; Charles E. Ford, PhD^†^; Jeffrey L. Probstfield, MD^§^; Barbara L. Beard, DO^‖^; Horia Marginean, MD,MS^¶^; Bruce P. Hamilton, MB,ChB^**^; Pamela S. Suhan, RN^††^; Barry R. Davis, MD, PhD^†^; on behalf of the ALLHAT Collaborative Research Group

Affiliations: ^*^Zablocki Veterans Affairs Medical Center, Milwaukee, WI; ^†^The University of Texas School of Public Health, Houston, TX; ^‡^Wake Forest University School of Medicine, Winston-Salem, NC; ^§^University of Washington Medical Center, Seattle, WA; ^‖^E.A. Conway Hospital, Monroe, LA; ^¶^Ottawa Hospital, Ottawa, Ontario; ^**^Veterans Affairs Medical Center, Baltimore, MD; ^††^The University of Toledo, Toledo, OH. Members of the ALLHAT Collaborative Research Group are listed in JAMA 2000;283:1973-1975.

Short title: Correlates of adherence

Corresponding author:

José-Miguel Yamal, PhD

Division of Biostatistics

Coordinating Center for Clinical Trials

1200 Pressler Street

Houston, TX 77030

Tel: +1 713 500 9566

Fax: +1 713 500 9530

Jose-Miguel.Yamal@uth.tmc.edu

Clinical Trial Registration: [www.clinicaltrials.gov](file:///\\SPHFiles\Home$\lpiller\My%20Documents\Abstracts%20and%20Publications\Williamson_Probstfield_GI_Bleed\OCT%202011\TITLE%20PAGE\www.clinicaltrials.gov), NCT00000542

Table S1. Baseline Characteristics by Continuous Visit Adherence*

|  | Visit Adherence | |
| --- | --- | --- |
| Baseline Characteristic | Proportion of completed visits | P-value |
| Gender, mean (SD) |  | <0.001 |
| Male (N=17343) | 0.803 (0.253) |  |
| Female (N=15395) | 0.750 (0.282) |  |
| Age Group (years), mean (SD) |  | <0.001 |
| 55-64 (N=14014) | 0.768 (0.269) |  |
| 65-74 (N=13176) | 0.789 (0.264) |  |
| ≥75 (N=5548) | 0.778 (0.274) |  |
| Race and Ethnicity, mean (SD) |  | <0.001 |
| Black (N=11572) | 0.739 (0.283) |  |
| White Hispanic (N=5146) | 0.689 (0.315) |  |
| White Non-Hispanic (16020) | 0.835 (0.225) |  |
| Ethnicity, mean (SD) |  | <0.001 |
| Non-Hispanic (N=26526) | 0.804 (0.244) |  |
| Hispanic (N=6212) | 0.667 (0.330) |  |
| Education |  | <0.001 |
| < High school (N=13215) | 0.759 (0.282) |  |
| High School (N=8700) | 0.800 (0.253) |  |
| Beyond high school (N=8609) | 0.809 (0.244) |  |
| Type II Diabetes, mean (SD) |  | <0.001 |
| Yes (N=11810) | 0.767 (0.276) |  |
| No (N=20928) | 0.785 (0.264) |  |
| Smoking, mean (SD) |  | <0.001 |
| Current (N=7121) | 0.769 (0.270) |  |
| Past (N=13212) | 0.802 (0.254) |  |
| Never (N=12403) | 0.758 (0.280) |  |
| Self-assessed baseline health, mean (SD) |  | <0.001 |
| Excellent (N=1813) | 0.819 (0.234) |  |
| Very good (N=7639) | 0.790 (0.270) |  |
| Good (N=14593) | 0.778 (0.269) |  |
| Fair (N=7440) | 0.764 (0.270) |  |
| Poor (N=917) | 0.735 (0.275) |  |
| Unknown (N=336) | 0.708 (0.283) |  |
| Practice type, mean (SD) |  | <0.001 |
| Private (N=9845) | 0.748 (0.283) |  |
| Group (N=6403) | 0.805 (0.250) |  |
| HMO (N=1150) | 0.596 (0.390) |  |
| Community Health (N=2663) | 0.767 (0.260) |  |
| University (N=3011) | 0.812 (0.240) |  |
| Other (N=2744) | 0.776 (0.257) |  |
| VSMC (N=5503) | 0.868 (0.187) |  |
| Unknown (N=1419) | 0.616 (0.301) |  |
| Baseline Medications, mean (SD) |  | <0.001 |
| On 1-2 drugs ≥ 2 months (N=28416) | 0.784 (0.266) |  |
| On 1-2 drugs < 2 months (N=1114) | 0.734 (0.293) |  |
| Currently untreated (N=3207) | 0.743 (0.278) |  |
| HDL Cholesterol < 35 mg/dl, mean (SD) |  | <0.001 |
| Yes (N=3829) | 0.809 (0.257) |  |
| No (N=28909) | 0.774 (0.269) |  |
| Aspirin use, mean (SD) |  | <0.001 |
| Yes (N=11737) | 0.814 (0.244) |  |
| No (N=20591) | 0.760 (0.278) |  |
| Geographic Region, mean (SD) |  | <0.001 |
| Northeast (N=4918) | 0.799 (0.253) |  |
| Midwest (N=5932) | 0.828 (0.231) |  |
| South (N=13624) | 0.778 (0.255) |  |
| West (N=3164) | 0.834 (0.216) |  |
| Canada (N=542) | 0.917 (0.115) |  |
| Puerto Rico/Virgin Islands (N=4558) | 0.635 (0.347) |  |
| Possible disability, mean (SD) |  | <0.001 |
| Yes (N=5836) | 0.828 (0.224) |  |
| No (N=26902) | 0.767 (0.276) |  |
| ASCVD, mean (SD) |  | <0.001 |
| Yes (N=11863) | 0.788 (0.267) |  |
| Sub-clinical (N=9029) | 0.783 (0.256) |  |
| No (N=11846) | 0.765 (0.278) |  |
| BMI, mean (SD) |  | 0.702 |
| ≤30 (N=19014) | 0.779 (0.270) |  |
| >30 (N=13624) | 0.778 (0.265) |  |
| Randomized treatment group, mean (SD) |  | <0.001 |
| Chlorthalidone (N=14975) | 0.783 (0.267) |  |
| Amlodipine (N=8875) | 0.783 (0.265) |  |
| Lisinopril (N=8888) | 0.766 (0.273) |  |

Abbreviations: ASCVD, atherosclerotic cardiovascular disease; BMI, body-mass index; HDL, high-density lipoprotein; HMO, health maintenance organization

^*^Visit Adherence= number of visits completed in the target windows divided by the number of expected visits (number of visits possible with perfect adherence).

Table S2. Baseline Characteristics by Medication and Visit Adherence, Truncated after Event^*^

|  | Medication Adherence^†^ | | | Visit Adherence^‡^ | | | |  |
| --- | --- | --- | --- | --- | --- | --- | --- | --- |
| Baseline Characteristic | N | Adequate Adherence | P-value | | N | Adequate Adherence | P-value | |
| Gender, n (%) |  |  | <0.001 | |  |  | <0.001 | |
| Male | 14,882 | 11,223 (75.4) |  |  | 15,805 | 11,632 (73.6) |  |  |
| Female | 13,133 | 9,499 (72.3) |  |  | 14,447 | 9,276 (64.2) |  |  |
| Age Group (years), n (%) |  |  | <0.001 | |  |  | <0.001 | |
| 55-64 | 12,260 | 9,118 (74.4) |  |  | 13,193 | 8,838 (67.0) |  |  |
| 65-74 | 11,175 | 8,343 (74.7) |  |  | 12,048 | 8,592 (71.3) |  |  |
| ≥75 | 4,580 | 3,261 (71.2) |  |  | 5,011 | 3,478 (69.4) |  |  |
| Race and Ethnicity, n (%) |  |  | <0.001 | |  |  | <0.001 | |
| Black | 9,917 | 6,952 (70.1) |  |  | 10,813 | 6,667 (61.8) |  |  |
| Non-Black Hispanic | 4,209 | 2,716 (64.5) |  |  | 4,964 | 2,741 (55.2) |  |  |
| Non-Black Non-Hispanic | 13,889 | 11,054 (79.6) |  |  | 14,475 | 11,490 (79.4) |  |  |
| Education, n (%) |  |  | <0.001 | |  |  | <0.001 | |
| < High school | 11,103 | 7,864 (70.8) |  |  | 12,181 | 8,021 (65.8) |  |  |
| High School | 7,539 | 5,735 (76.1) |  |  | 8,006 | 5,793 (72.4) |  |  |
| Beyond high school | 7,548 | 5,835 (77.3) |  |  | 7,993 | 5,951 (74.5) |  |  |
| Type II Diabetes, n (%) |  |  | 0.840 | |  |  | 0.001 | |
| Yes | 9,961 | 7,375 (74.0) |  |  | 10,837 | 7,367 (68.0) |  |  |
| No | 18,054 | 13,347 (73.9) |  |  | 19,415 | 13,541 (69.7) |  |  |
| Smoking, n (%) |  |  | <0.001 | |  |  | <0.001 | |
| Current | 6,071 | 4,394 (72.4) |  |  | 6,560 | 4,407 (67.2) |  |  |
| Past | 11,297 | 8,573 (75.9) |  |  | 12,055 | 8,832 (73.3) |  |  |
| Never | 10,647 | 7,755 (72.8) |  |  | 11,636 | 7,669 (65.9) |  |  |
| Self-assessed baseline health, n (%) |  |  | <0.001 | |  |  | <0.001 | |
| Excellent | 1,626 | 1,256 (77.2) |  |  | 1,701 | 1,269 (74.6) |  |  |
| Very good | 6,606 | 4,999 (75.7) |  |  | 7,124 | 5,148 (72.3) |  |  |
| Good | 12,498 | 9,257 (74.1) |  |  | 13,550 | 9,337 (68.9) |  |  |
| Fair | 6,284 | 4,455 (70.9) |  |  | 6,779 | 4,490 (66.2) |  |  |
| Poor | 719 | 538 (74.8) |  |  | 788 | 488 (61.9) |  |  |
| Unknown | 282 | 217 (77.0) |  |  | 310 | 176 (56.8) |  |  |
| Practice type, n (%) |  |  | <0.001 | |  |  | <0.001 | |
| Private | 8,369 | 5,628 (67.2) |  |  | 9,283 | 5,978 (64.4) |  |  |
| Group | 5,516 | 4,417 (80.1) |  |  | 5,849 | 4,305 (73.6) |  |  |
| HMO | 838 | 670 (80.0) |  |  | 1,112 | 548 (49.3) |  |  |
| Community Health | 2,359 | 1,722 (73.0) |  |  | 2,495 | 1,633 (65.5) |  |  |
| University | 2,614 | 1,927 (73.7) |  |  | 2,734 | 2,073 (75.8) |  |  |
| Other | 2,333 | 1,821 (78.1) |  |  | 2,530 | 1,691 (66.8) |  |  |
| VAMC | 4,748 | 3,643 (76.7) |  |  | 4,889 | 4,126 (84.4) |  |  |
| Unknown | 1,238 | 894 (72.2) |  |  | 1,360 | 554 (40.7) |  |  |
| Baseline Medications, n (%) |  |  | <0.001 | |  |  | <0.001 | |
| On 1-2 drugs ≥ 2 months | 24,284 | 18,116 (74.6) |  |  | 26,201 | 18,374 (70.1) |  |  |
| On 1-2 drugs < 2 months | 956 | 669 (70.0) |  |  | 1,041 | 655 (62.9) |  |  |
| Currently untreated | 2,775 | 1,937 (69.8) |  |  | 3,009 | 1,879 (62.4) |  |  |
| HDL Cholesterol < 35 mg/dl, n (%) |  |  | <0.001 | |  |  | <0.001 | |
| Yes | 3,297 | 2,577 (78.2) |  |  | 3,505 | 2,667 (76.1) |  |  |
| No | 24,718 | 18,145 (73.4) |  |  | 26,747 | 18,241 (68.2) |  |  |
| Aspirin use, n (%) |  |  | <0.001 | |  |  | <0.001 | |
| Yes | 9,914 | 7,682 (77.5) |  |  | 10,548 | 7,961 (75.5) |  |  |
| No | 17,768 | 12,803 (72.1) |  |  | 19,319 | 12,746 (66.0) |  |  |
| Geographic Region, n (%) |  |  | <0.001 | |  |  | <0.001 | |
| Northeast | 4,270 | 3,206 (75.1) |  |  | 4,518 | 3,294 (72.9) |  |  |
| Midwest | 5,152 | 3,876 (75.2) |  |  | 5,352 | 4,161 (77.7) |  |  |
| South | 11,764 | 8,925 (75.9) |  |  | 12,542 | 8,494 (67.7) |  |  |
| West | 2,811 | 2,094 (74.5) |  |  | 2,918 | 2,280 (78.1) |  |  |
| Canada | 487 | 388 (79.7) |  |  | 491 | 465 (94.7) |  |  |
| Puerto Rico/Virgin Islands | 3,531 | 2,233 (63.2) |  |  | 4,431 | 2,214 (50.0) |  |  |
| Possible disability, n (%) |  |  | 0.965 | |  |  | <0.001 | |
| Yes | 5,135 | 3,797 (73.9) |  |  | 5,417 | 4,176 (77.1) |  |  |
| No | 22,880 | 16,925 (74.0) |  |  | 24,835 | 16,732 (67.4) |  |  |
| ASCVD, n (%) |  |  | <0.001 | |  |  | <0.001 | |
| Yes | 9,828 | 7,538 (76.7) |  |  | 10,665 | 7,606 (71.3) |  |  |
| Sub-clinical | 7,846 | 5,452 (69.5) |  |  | 8,336 | 5,692 (68.3) |  |  |
| No | 10,341 | 7,732 (74.8) |  |  | 11,251 | 7,610 (67.6) |  |  |
| BMI, n (%) |  |  | 0.122 | |  |  | 0.044 | |
| ≤30 | 16,217 | 11,937 (73.6) |  |  | 17,545 | 12,210 (69.6) |  |  |
| >30 | 11,721 | 8,724 (74.4) |  |  | 12,621 | 8,646 (68.5) |  |  |
| Randomized treatment group, n (%) |  |  | 0.112 | |  |  | <0.001 | |
| Chlorthalidone | 12,900 | 9,565 (74.1) |  |  | 13,928 | 9,757 (70.1) |  |  |
| Amlodipine | 7,652 | 5,702 (74.5) |  |  | 8,215 | 5,770 (70.2) |  |  |
| Lisinopril | 7,463 | 5,455 (73.1) |  |  | 8,109 | 5,381 (66.4) |  |  |

Abbreviations: ASCVD, atherosclerotic cardiovascular disease; BMI, body-mass index; HDL, high-density lipoprotein; HMO, health maintenance organization

*Excluding visits after a participant developed cancer (except non-melanoma skin cancer) or end stage renal disease (ESRD) or had a new CVD event, including stroke, myocardial infarction, coronary artery revascularization, angina, congestive heart failure, or peripheral vascular disease.

^†^Medication Adherence is a dichotomous variable defined as poor adherence if the participant reported taking less than 80% of their step 1 medication at any visit and as good if they reported taking 80% or more of this medication at all visits. The percentage of total participants in each baseline category that were categorized as adherent is presented.

^‡^Visit Adherence is a dichotomous variable defined as poor if the participant attended less than 80% of their scheduled visits within the study window and as adequate if they attended at least 80% of their scheduled visits within the study window. The percentage of total participants in each baseline category that were categorized as adherent is presented.

Table S3. Independent Effect of Baseline Characteristics on Visit Adherence, Truncated after Event^*^

|  | Odds Ratio (95% CI) - Multiple Logistic Regression | | | |
| --- | --- | --- | --- | --- |
| Baseline Characteristic | (Full Model)  (N = 27,941) | P-value | (Reduced Model)  (N = 27,941) | P-value |
| Male | 1.08 (1.02 – 1.15) | 0.013 | 1.08 (1.02 – 1.15) | 0.010 |
| Age (years) (55-64 is the reference group) |  |  |  |  |
| 65-74 | 1.42 (1.33 – 1.52) | <0.001 | 1.41 (1.31 – 1.50) | <0.001 |
| ≥75 | 1.36 (1.25 – 1.49) | <0.001 | 1.34 (1.23 – 1.46) | <0.001 |
| Race and Ethnicity (Black is reference group)† |  |  |  |  |
| Non-Black Hispanic | 0.94 (0.87 – 1.02) | 0.149 | 0.94 (0.87 – 1.02) | 0.125 |
| Non-Black Non-Hispanic | 2.09 (1.95 – 2.23) | <0.001 | 2.12 (1.98 – 2.26) | <0.001 |
| Education (High school is reference group) |  |  |  |  |
| < High school | 0.95 (0.89 – 1.01) | 0.120 |  |  |
| Beyond high school | 1.01 (0.94 – 1.09) | 0.727 |  |  |
| Has Diabetes | 1.00 (0.94 – 1.07) | > 0.99 |  |  |
| Smoking (Current is reference group) |  |  |  |  |
| Past | 1.16 (1.07 – 1.25) | <0.001 | 1.16 (1.08 – 1.25) | <0.001 |
| Never | 1.08 (1.00 – 1.17) | 0.059 | 1.08 (1.00 – 1.17) | 0.039 |
| Self-assessed baseline health (Excellent is reference group) |  |  |  |  |
| Very good | 0.93 (0.81 – 1.06) | 0.256 | 0.93 (0.81 – 1.05) | 0.243 |
| Good | 0.91 (0.80 – 1.03) | 0.123 | 0.90 (0.80 – 1.02) | 0.101 |
| Fair | 0.85 (0.74 – 0.97) | 0.014 | 0.84 (0.73 – 0.95) | 0.008 |
| Poor | 0.59 (0.49 – 0.72) | <0.001 | 0.58 (0.48 – 0.71) | <0.001 |
| Unknown | 0.70 (0.46 – 1.05) | 0.084 | 0.69 (0.46 – 1.04) | 0.078 |
| Practice type (Private is the reference group) |  |  |  |  |
| Group | 1.19 (1.09 – 1.29) | <0.001 | 1.19 (1.09 – 1.29) | <0.001 |
| HMO | 0.53 (0.47 – 0.61) | <0.001 | 0.53 (0.47 – 0.61) | <0.001 |
| Community Health | 1.07 (0.97 – 1.19) | 0.184 | 1.07 (0.96 – 1.18) | 0.205 |
| University | 1.64 (1.47 – 1.83) | <0.001 | 1.64 (1.47 – 1.83) | <0.001 |
| Other | 1.10 (0.99 – 1.22) | 0.077 | 1.10 (0.99 – 1.22) | 0.076 |
| VSMC | 2.03 (1.83 – 2.24) | <0.001 | 2.03 (1.84 – 2.25) | <0.001 |
| Unknown | 0.40 (0.35 – 0.46) | <0.001 | 0.40 (0.35 – 0.45) | <0.001 |
| Baseline Medications (Currently untreated is the reference group) |  |  |  |  |
| On 1-2 drugs < 2 months | 1.42 (1.30 – 1.55) | <0.001 | 1.42 (1.30 – 1.55) | <0.001 |
| On 1-2 drugs ≥ 2 months | 1.08 (0.92 – 1.27) | 0.349 | 1.08 (0.92 – 1.27) | 0.335 |
| HDL Cholesterol < 35 mg/dl | 1.13 (1.03 – 1.24) | 0.009 | 1.14 (1.04 – 125) | 0.007 |
| Taking aspirin | 1.20 (1.13 – 1.28) | <0.001 | 1.21 (1.13 – 1.28) | <0.001 |
| Possible disability | 2.08 (1.91 – 2.27) | <0.001 | 2.08 (1.91 – 2.26) | <0.001 |
| ASCVD (No ASCVD is the reference group) |  |  |  |  |
| Yes | 0.94 (0.87 – 1.01) | 0.084 | 0.94 (0.87 – 1.00) | 0.053 |
| Sub-clinical | 1.07 (0.99 – 1.15) | 0.101 | 1.07 (0.99 – 1.14) | 0.073 |
| BMI > 30 | 1.01 (0.95 - 1.07) | 0.746 |  |  |
| Decile of clinic enrollment volume | 1.02 (1.01 – 1.03) | 0.001 | 1.02 (1.01 – 1.03) | 0.003 |
| Clinic size | 1.02 (1.01 – 1.03) | 0.001 | 1.02 (1.01 – 1.03) | 0.003 |
| Randomized treatment group (Chlorthalidone is the reference group) |  |  |  |  |
| Amlodipine | 1.00 (0.94 – 1.07) | 0.897 | 1.00 (0.94 – 1.07) | 0.914 |
| Lisinopril | 0.84 (0.78 – 0.89) | <0.001 | 0.84 (0.78 – 0.89) | <0.001 |

Abbreviations: ASCVD, atherosclerotic cardiovascular disease; BMI, body-mass index; HDL, high-density lipoprotein; HMO, health maintenance organization

^*^Visit Adherence is a dichotomous variable defined as poor if the total patient record of “% visit adherence” was < 80% and adequate adherence if adherence was ≥ 80%.

Table S4. Independent Effect of Baseline Characteristics on Medication Adherence, Truncated after Event^*^

|  | Odds Ratio (95% CI) - Multiple Logistic Regression | | | |
| --- | --- | --- | --- | --- |
| Baseline Characteristic | Full Model  (N = 25,979) | P-value | Reduced Model  (N = 25,979) | P-value |
| Adequate Visit Adherence | 1.03 (0.96 – 1.10) | 0.419 |  |  |
| Male | 1.02 (0.96 – 1.09) | 0.499 |  |  |
| Age (years) (55-64 is the reference group) |  |  |  |  |
| 65-74 | 0.92 (0.86 – 0.99) | 0.035 | 0.92 (0.86 – 0.99) | 0.034 |
| ≥75 | 0.80 (0.73 – 0.88) | <0.001 | 0.80 (0.73 – 0.88) | <0.001 |
| Race and Ethnicity (Black is the reference group) † |  |  |  |  |
| Non-Black Hispanic | 0.75 (0.69 – 0.82) | <0.001 | 0.75 (0.69 – 0.82) | <0.001 |
| Non-Black Non-Hispanic | 1.51 (1.41 – 1.62) | <0.001 | 1.52 (1.42 – 1.63) | <0.001 |
| Education (High school is the reference group) |  |  |  |  |
| < High school | 0.91 (0.85 – 0.98) | 0.010 | 0.91 (0.85 – 0.98) | 0.009 |
| Beyond high school | 1.01 (0.93 – 1.09) | 0.897 | 1.01 (0.93 - 1.09) | 0.864 |
| Has diabetes | 1.00 (0.93 – 1.07) | 0.895 |  |  |
| Smoking (Current is the reference group) |  |  |  |  |
| Past | 1.16 (1.07 – 1.26) | <0.001 | 1.17 (1.08 – 1.27) | <0.001 |
| Never | 1.15 (1.06 – 1.25) | <0.001 | 1.16 (1.07 – 1.25) | <0.001 |
| Self-assessed baseline health (Excellent is the reference group) |  |  |  |  |
| Very good | 0.95 (0.83 – 1.08) | 0.420 | 0.95 (0.83 – 1.08) | 0.421 |
| Good | 0.93 (0.82 – 1.06) | 0.250 | 0.93 (0.82 – 1.05) | 0.242 |
| Fair | 0.83 (0.72 – 0.95) | 0.007 | 0.83 (0.72 – 0.95) | 0.006 |
| Poor | 0.98 (0.79 – 1.21) | 0.832 | 0.97 (0.79 – 1.20) | 0.791 |
| Unknown | 0.95 (0.61 – 1.49) | 0.826 | 0.95 (0.61 – 1.49) | 0.824 |
| Practice type (Private is the reference group) |  |  |  |  |
| Group | 1.65 (1.51 – 1.80) | <0.001 | 1.65 (1.51 – 1.81) | <0.001 |
| HMO | 2.01 (1.68 – 2.42) | <0.001 | 2.01 (1.68 – 2.42) | <0.001 |
| Community Health | 1.27 (1.14 – 1.42) | <0.001 | 1.27 (1.14 – 1.42) | <0.001 |
| University | 1.27 (1.14 – 1.41) | <0.001 | 1.27 (1.14 – 1.41) | <0.001 |
| Other | 1.73 (1.54 – 1.95) | <0.001 | 1.73 (1.54 – 1.95) | <0.001 |
| VSMC | 1.28 (1.16 – 1.41) | <0.001 | 1.30 (1.19 – 1.42) | <0.001 |
| Unknown | 1.54 (1.33 – 1.78) | <0.001 | 1.53 (1.33 – 1.77) | <0.001 |
| Baseline Medications (Currently untreated is the reference group) |  |  |  |  |
| On 1-2 drugs < 2 months | 1.25 (1.14 – 1.37) | <0.001 | 1.25 (1.14 – 1.37) | <0.001 |
| On 1-2 drugs ≥ 2 months | 1.10 (0.92 - 1.30) | 0.300 | 1.10 (0.93 – 1.30) | 0.288 |
| HDL Cholesterol < 35 mg/dl | 1.06 (0.96 – 1.17) | 0.240 |  |  |
| Taking aspirin | 1.15 (1.08 – 1.23) | <0.001 | 1.16 (1.09 – 1.23) | <0.001 |
| Possible disability | 0.92 (0.84 – 1.00) | 0.057 | 0.92 (0.85 – 1.01) | 0.071 |
| ASCVD (No ASCVD is the reference group) |  |  |  |  |
| Yes | 0.99 (0.92 – 1.07) | 0.829 | 0.99 (0.92 – 1.06) | 0.697 |
| Sub-clinical | 0.78 (0.72 – 0.85) | <0.001 | 0.78 (0.73 – 0.84) | <0.001 |
| BMI > 30 | 1.03 (0.97 – 1.09) | 0.400 |  |  |
| Decile of clinic enrollment volume | 1.05 (1.04 – 1.06) | <0.001 | 1.05 (1.04 – 1.06) | <0.001 |
| Randomized treatment group (Chlorthalidone is the reference group) |  |  |  |  |
| Amlodipine | 1.01 (0.94 – 1.08) | 0.805 |  |  |
| Lisinopril | 0.94 (0.88 – 1.01) | 0.077 |  |  |

Abbreviations: ASCVD, atherosclerotic cardiovascular disease; BMI, body-mass index; HDL, high-density lipoprotein; HMO, health maintenance organization

^*^Medication Adherence is a dichotomous variable defined as poor if they reported taking less than 80% of their step 1 medication at any visit and as adequate if they reported taking 80% or more of this medication at all visits.
